# Further Observations on the Arrest of Puerperal Fever by Inhibition of the Pulse

**Published:** 1897-03-20

**Authors:** Glynn Whittle

**Affiliations:** Senior Physician to the Liverpool Lying-In Hospital.


### FURTHER OBSERVATIONS ON THE ARREST OF PUERPERAL FEVER BY INHIBITION OF THE PULSE.

By Glynn Whittle, M.A., M.D., Cantab, M.R.C.P., Senior Physician to the Liverpool Lying-in Hospital.

The chief defect of former midwifery teaching was undoubtedly the omission to explain the difficulties which may attend the procedure of administering the uterine douche, a neglect to be deplored as having led to the needless loss of countless lives, and a matter therefore whose importance I take this opportunity of publicly emphasising.

The student's attention was never sufficiently directed to the pathology and pathological anatomy of the cervix uteri and its varying conditions in puerperal septicæmia. The unfortunate result has too often been inadequate knowledge of the subject on the part of the practitioner.

Every experienced expert in midwifery has probably met with instances of practitioners who, though in other respects skilful and even able obstetricians, are not reliable as to their ideas and methods of administering a uterine douche. In some cases they are as absolutely convinced that they have syringed the cavity of the uterus, as it is unfortunately true that not a drop of the fluid has found its way inside it.

The cervix uteri and its conditions in fever would alone constitute an ample subject for discussion, but it is a topic which on the present occasion I do not propose to refer to further than stating that the operation of the uterine douche may be attended with difficulty, and that it is by no means a procedure which it is always within the power of a practitioner to forthwith readily perform.

Some years ago I published a list of cases of puerperal fever, the principal feature of the treatment being large doses of digitalis or aconite. In all the patients the marked fall in the pulse was attributed to the toxic action of the remedy, and in half of the cases the influence of the drug on the circulation was proved by the irregular and intermittent character of the pulse during convalescence.

If any form of severe local inflammation complicates the septicæmia, inhibition of the pulse, though it is useful as a measure of help, cannot be regarded as the chief part of the treatment, which must of course be directed to the immediate relief of the inflamed part.

It is difficult for puerperal septicæmia to prove fatal without first disorganising the action of the heart, while the rate of the pulse, maintained for a sufficiently long time at a high degree of acceleration, is generally an essential preliminary of such complete disturbance of the circulation. This loss of control of the circulation next occasions or aggravates that grave physical sign—delirium. Hitherto the healthy cellular elements of the brain had successfully held in check the invading forces of the sepsis, but, owing to the failure of the circulation, now show signs of being overcome by them. Even if, what would otherwise be a favourable fall in temperature, now occurs, the patient may nevertheless die from the brain being poisoned past recovery; for the conditions effecting dissolution by no means necessarily include hyper-pyrexia. Therefore, whether for the sake of the brain or to prevent hyper-pyrexia, the disease must not be allowed to get the upper hand of the circulation.

In earlier practice, finding many cases in which the application of the uterine douche was attended with difficulty, the view of the pathology, which I have here stated, led me to treat the fever with heroic doses of aconite, but I soon began to use digitalis instead, as a drug which could be more safely pushed. I experimented with both drugs in doses larger than I would now give, and which would have been risky if it had not been for the extreme care with which I watched their results; but good luck followed me, and I was ultimately able to arrive at a dose range—both safe and efficacious—without experiencing the painful lesson of ever fatally poisoning a patient.

It has been claimed for surgery that the antiseptic uterine douche should be the sole treatment of this form of septicæmia. I desire, however, to distribute equitably between medicine and surgery their respective shares in governing the matter in dispute. One of the medical lessons which I have learnt through watching the course of the disease and observing the effects of digitalis is perceiving its indirect action as an anti-spasmodic to tonic contraction of the os uteri internum. The drop in the pulse, accompanied by the characteristic improvement in its beat, indicating that the administration of digitalis is successfully accomplishing its expected work, is followed more tardily by a satisfactory abatement in the temperature. This circumstance points to the conclusion that it is through its influence on the circulation that the drug favours a relaxation of the tight and dangerous spasm of the sphincter at the os uteri internum, and in this way permits a freer drainage of the lochia, and consequent fall in the temperature.

The general system of the treatment is to give 10 m. of tincture of digitalis every three hours, the dose being doubled or trebled if necessary at any time according to the indications of the pulse.

In the following ten cases the uterine douche was used only sparingly, viz., once in each of the last three, and in the first seven patients not at all. The first three indicate the minimum amount of tincture of digitalis likely to be of any inhibitory service, while the tenth represents about the maximum amount of the drug which may be safely given with judgment, courage, and caution.

1. This was a case of septicæmia in a young primipara, the pulse on the third day rising to 146 and the temperature to 103·4. Ten m. of tincture of digitalis were given every three hours for a few days. After commencement of the treatment the patient never got worse; so the uterine douche was not given.

2. This case was similar to Number 1, but the symptoms were less severe.

3. This had been a specially anxious and troublesome primiparous labour, a very quick pulse and a certain amount of fever having preceded a tardy delivery. She was placed under treatment by tincture of digitalis, and took one fluid ounce in six days, when the symptoms abated.

4. This was an elderly primipara, delivered by forceps. She had a slow pulse, about 60, and the incipient fever was consequently easily nipped in the bud. About forty-eight hours after delivery the lochia became suppressed, the temperature rising to 101 and the pulse to 90. An 8 ounce mixture containing 3 fluid drachms of tincture of digitalis was ordered, half a fluid ounce every three hours. The next day, the symptoms being unchanged, a treble dose was given, and after two more ordinary doses the lochia flowed freely, and the feverish symptoms disappeared.

5. This was a multipara in whom the feverish pulse rapidly rose as high as 152, 10 m. of tincture of digitalis being ordered every three hours, but the dose being doubled on three occasions. The signs soon became milder, and abated altogether in the course of a few days.

6. This case was similar to Number 5, but occurred after instrumental delivery in a rigid primipara.

7. The symptoms here followed a severe instrumental labour in a rigid primipara. I gave 30 m. of tincture of digitalis, the pulse being 138, and ordered 10 m. every three hours, to be increased to 20 if the pulse was found to be over 130. Four such extra doses were required in all, and on the sixth day recovery was established, but with an intermittent pulse—the temporary effect of the drug.

8. This case commenced very suddenly, the pulse rising to 140 and the temperature to 103·4. As the patient lived in Rock Ferry, and I could not make very frequent visits, I douched the uterus to be on the safe side, and then gave tincture of digitalis, administering a fluid ounce in five days when favourable resolution occurred.

9. This was an excessively severe instrumental case in deformed rickety pelvis. A still-born child was ultimately delivered with the long German forceps, and at the conclusion of labour, on account of the bruising of the soft parts, I douched the uterus. Subsequently fever appeared, and rapidly developed, the pulse rising to 140 and the temperature to 102. Ten m. of tincture of digitalis were ordered every three hours, 20 m. to be given when the pulse was found to be over 130. Only three such extra doses were required in all, and the course of the fever was speedily arrested.

10. This patient, a delicate lady, became alarmingly ill on the third day, a mental shock being the chief cause. Slight delirium was present. The temperature being 104·6 and the pulse 146, I douched the uterus, and prescribed tincture of digitalis, which was continued for five days. The drug at once checked the pulse, which descended in direct proportion as the digitalis was more freely given, but ascended again as the drug was eased off until the end of five days' treatment, when the pulse became normal, and the fever soon abated. In these five days the patient swallowed the large amount of 12 fluid drachms and 30 m. In this case I did attempt to douche the uterus a second time, but encountered spasm, and, knowing that manipulative resistance to spasm is dangerous to patients in feverish crises, abandoned the attempt and relied on the drug and my experience in how to administer it.

In all the cases fresh tincture was obtained from one of the leading firms, the reliability of the chemist and purity of the drug being vital points for securing inhibition of the pulse.

